# Medical aesthetic awareness among public in Malaysia and the factors that may influence it: A cross-sectional study

**DOI:** 10.51866/oa.458

**Published:** 2024-07-15

**Authors:** Yong Teck Hoang Allan, Kwong Fai Ng Richard, Firuz Nur Dianah, Jagathisan Prathishaa, Samari Syafiqa Nur 'Ukail, Hatah Ernieda Md, Abdul Rashid Muhammad Farhan, Mohd Shahrin Ungku Mohd Zaman, Hanim Ismail Adibah

**Affiliations:** 1 BPharm(Hons), MPharm(Clin), PhD, Faculty of Pharmacy, University Kebangsaan Malaysia, Jalan Raja Muda Abdul Aziz, Kuala Lumpur, Malaysia. Email: ernieda@ukm.edu.my; 2 MBBS, Post Grad DipDerm, AAAM, ABAARM., W Clinic, Ground floor of No.39, Wisma CUIR, Jalan Maarof, Bangsar, Wilayah Persekutuan, Kuala Lumpur, Malaysia.; 3 MBBS, MBA, MMIM, Clinic RX, Wisma TTGU, Jalan Maarof, Bangsar, Wilayah Persekutuan, Kuala Lumpur, Malaysia.; 4 MBBCh, Klinik Fiana Antara Gapi Dr Sofian & Rakan-rakan, No 3A-G, Jalan Gapimas 1, Laman Gapimas, Serendah, Selangor, Malaysia.; 5 MD, Faculty of Medicine and Health Sciences, University Malaysia Sarawak, Jalan Datuk Mohammad Musa, Kota Samarahan, Sarawak, Malaysia.; 6 B.Comm, Faculty of Applied Communication, Multimedia University, Cyberjaya, Selangor, Malaysia.; 7 Bsc, USMARI Research and Innovation Centre, S68-1, Red Carpet Avenue, Encorp Strand Mall, Kota Damansara PJU 5/22, Petaling Jaya, Selangor, Malaysia.; 8 MD, USMARI Research and Innovation Centre, S68-1, Red Carpet Avenue, Encorp Strand Mall, Kota Damansara PJU 5/22,Petaling Jaya, Selangor, Malaysia.; 9 MD, M.Med (Fam. Med), Family Medicine Department, Faculty of Medicine and Health Sciences, Universiti Putra Malaysia (UPM). Serdang, Selangor.

**Keywords:** Medical aesthetics, Survey, Malaysia, Awareness, Physical attractiveness

## Abstract

**Introduction:**

Medical aesthetic practice is growing rapidly in Malaysia due to rising market demand, yet public understanding of these practices remains limited. This study evaluated the awareness and attitudes towards medical aesthetics among Malaysians.

**Methods:**

A cross-sectional survey was conducted among individuals aged > 18 years old in Malaysia from December 2021 to May 2022. The survey was distributed both online and in public settings. The survey comprised of respondents’ sociodemographic, perception of physical attractiveness, knowledge and attitude towards medical aesthetic practices. The factors influencing respondents’ attitudes towards medical aesthetic practices in the country were analysed using binary logistic regression, with the significance level set at P<0.05.

**Results:**

A total of 382 respondents participated in this study with average age of 30.81 (±9.38) years, ranging from 18 to 68 years. The majority were women (77.5%), Malay (53.7%) and Muslim (57.1%). Although most respondents had no prior experience in medical aesthetic treatment (68.1%), 76.2% respondents demonstrated good knowledge and 70.2% had positive attitude towards these services. Additionally, 53.9% of the respondents highly valued the importance of physical attractiveness. The Buddhists and Hindus exhibited more positive attitude than the Muslims. Conversely, those who placed higher importance on physical attractiveness were 0.5 times less likely to develop a positive attitude towards medical aesthetic services.

**Conclusion:**

In conclusion, despite limited experience in medical aesthetic treatments and practice, most Malaysians possess good knowledge and positive attitudes towards medical aesthetics, indicating a growing interest and potential willingness to consider these services for enhancing their appearance.

## Introduction

Medical aesthetic procedures represent a relatively new field of medical practice involving various interventions that revise or alter the appearance, colour, texture, structure or position of bodily features, which most people would otherwise consider to be within the broad range of ‘normal’.^1^ It could include anything from minor cosmetic treatments to physical augmentation surgery as well as anti-ageing procedures.^2^ The Malaysian Medical Council defines medical aesthetic practice as an area of medical practice that embraces multidisciplinary modalities dedicated to creating a harmonious physical and psychological balance through evidence-based non-invasive, minimally invasive and invasive treatment modalities. These modalities focus on altering appearance in accordance with patients’ goals and are conducted by registered medical practitioners.^3^

Recently, there has been a notable growing demand for medical aesthetic treatments and procedures in Malaysia.^4^ However, recent medical aesthetic reports have highlighted concerning outcomes such as deformity and even mortality as a result of cosmetic procedures performed by unqualified and unlicensed practitioners. For instance, a tragic case of a girl passing away following breast augmentation oflered by an unqualified practitioner at a beauty centre has been reported, emphasising a significant lack of public awareness in Malaysia about the safe use of medical aesthetic treatments or procedures.^5^ Many consumers are likely unaware that medical aesthetic treatments or operations, as opposed to beauty salon or cosmetic medispa procedures, should be obtained only from facilities operated by a licensed medical aesthetician.^4^ Accordingly, the Ministry of Health (MOH) Malaysia created regulations on medical aesthetic practice to provide appropriate rules and guidelines. These regulations require all medical professionals to demonstrate their proficiency in the field and undergo rigorous evaluation before being granted a Letter of Credentialing and Privileging (LCP) in medical aesthetic practice. Despite the introduction of the LCP, there remains uncertainty regarding the public’s ability to differentiate between licensed medical aesthetic clinics and beauty centres. Hence, they are encouraged to verify all necessary certificates and credentials before undergoing any aesthetic treatment.^4^

In addition to raising medical aesthetic awareness, it is also critical for medical aesthetic practitioners to comprehend their customers’ perception of beauty to effectively advise and address their needs. The previous study by Redaelli et al. involving 460 respondents across Colombia, Russia, Thailand, Turkey, the United Arab Emirates and Malaysia found that certain physical key features of attractiveness and a healthy lifestyle were important for graceful ageing.^6^ Respondents had different perspectives on the importance of physical attributes, emotional well-being, internal beauty and self-confidence. Further, those who had undergone medical aesthetic treatments tended to express higher levels of satisfaction than those who had not.

While Redaelli et al.’s study offers insights into global perspectives on beauty, attitudes and experiences, including those in Malaysia, its limited sample size prevents the generalisation of conclusions to the broader Malaysian community.^6^ Given the potential disparity between the perceived ideals of facial beauty among medical aesthetic practitioners and the perspectives of their patients, it becomes crucial to investigate the beauty perceptions of patients or the public.^7^ Thus, this study aimed to assess public awareness, encompassing the perceived significance of physical attractiveness and the knowledge and attitudes towards medical aesthetic services in Malaysia. Assessing public awareness of medical aesthetic services in Malaysia is crucial in promoting informed decision-making, enhancing healthcare planning, mitigating risks, improving provider—patient dynamics, ensuring cultural sensitivity and laying the groundwork for future developments in the field.

## Methods

This study conducted a cross-sectional survey from December 2021 to May 2022 among the general public in Malaysia. The survey was disseminated through various online platforms including Facebook, Instagram, WhatsApp and Telegram and several public groups on Facebook such as Komuniti Selangor, Komuniti Johor Bahru and Komuniti Kedah. Respondents were further encouraged to share the survey link with their contacts, facilitating a broader reach and engagement within the target audience. Additionally, face-to-face survey distribution was conducted in public areas, including train and bus stations within Klang Valley, Selangor. Individuals who were aged >18 years, were Malaysian citizens and either had or had not received medical aesthetic treatment were included in the study. Conversely, individuals who had cognitive impairment, such as dementia or Alzheimer’s disease; had terminally ill diseases; were unable to provide informed consent; or were unable to understand Malay or English were excluded from this study. Respondents were provided with information about the study objectives and their rights to participate in the study through a study information sheet. They were also required to provide informed consent upon agreeing to participate in this study. For the online survey, respondents were informed that completing and submitting the questionnaire served as an indication of their consent to participate in the study. Participation in this study was voluntary, and no reimbursement was provided for respondents’ involvement.

The survey was developed internally based on previous literature and insights from expert opinions in the field.^8-11^ It consisted of four sections that focused on the following: A) sociodemographic and treatment characteristics, B) perceived significance of being physically attractive, C) knowledge of medical aesthetic services and D) attitude towards medical aesthetic services in Malaysia. Section A collected respondents’ sociodemographic and treatment characteristics, such as age, sex, marital status, race, religion, residing state and location, educational level, occupation, monthly income and previous experience with medical aesthetic services. Section B consisted of nine statements that explored the perceptions of the importance of being physically attractive. Section C comprised 10 statements that assessed the knowledge of medical aesthetic practices in Malaysia and six statements that evaluated the preferred place or setting for seeking specific medical aesthetic treatment. The response options for Section C included ‘true’, ‘false’ and ‘not sure’, with a score of 1 given for ‘true’ and 0 for ‘false’ or ‘not sure’. The total score for Section C was 10. Through a mean cut-off point, respondents scoring ≥6 were categorised as having good knowledge and those scoring <6 as having poor knowledge.^12^ Conversely, Section D utilised a 5-point Likert scale, with response options of ‘strongly agree’, ‘agree’, ‘neutral’, ‘disagree’ and ‘strongly disagree’. This section assessed the attitude towards medical aesthetic treatments in Malaysia through 10 statements related to the medical aesthetic field. Respondents were assigned scores ranging from 1 to 5 for their responses, with negative scoring applied to questions framed negatively. Through a mean cut-off point, respondents scoring ≥27 were categorised as having positive perceptions and those scoring <27 as having negative perceptions towards medical aesthetic practices in Malaysia.

A pilot study was conducted among 30 respondents to evaluate the content validity and reliability of the survey. All questions in the survey were reviewed and assessed by an expert team comprising three academicians and two medical aesthetic practitioners. Based on the pilot study findings, questions that were not clear were revised accordingly. The results showed Cronbach’s alpha values of 0.951, 0.724 and 0.858 for Sections B, C and D, respectively. Conversely, the item-level content validity index (I-CVI) and scale-level content validity index (S-CVI) were calculated to assess the relevancy of the questions judged by the expert team. The results revealed an I-CVI of 0.75–1 for each section. Conversely, the S-CVI was 0.89, 0.84 and 0.83 for Sections B, C and D, respectively, indicating that the survey was reliable and excellent.

Data were analysed descriptively and inferentially using the Statistical Package for the Social Sciences version 23.0(IBM SPSS Statistics, New York, United states). The descriptive analysis involved the presentation of respondents’ sociodemographic and treatment characteristics and scores on knowledge, perceptions and attitudes towards medical aesthetic treatments in Malaysia. Descriptive statistics such as frequencies, percentages, means, standard deviations (SDs) and medians were used where applicable. For the inferential analysis, backward binary logistic regression was utilised to assess the factors influencing respondents’ attitudes towards medical aesthetic practices in Malaysia. Prior to the analysis, a univariate test was conducted to select significant factors (P<0.25) for inclusion in the final model. In the final model analysis, factors with a P-value of <0.05 were considered statistically significant.

## Results

A total of 382 respondents participated in this study. The respondents’ mean age was 30.81 (SD=9.38) years, with an age range of 18–68 years. The majority of the respondents were women (n=296, 77.5%), single (n=242, 63.4%), Malay (n=205, 53.7%) and Muslim (n=218, 57.1%). Most respondents were residing in cities (n=289, 75.7%) and had attained up to a bachelor’s degree as their highest educational level (n=318, 83.3%). The majority were employed (n=237, 62.0%) and had an income of <RM 2500 (n=156, 40.8%). Additionally, most respondents had never received or experienced aesthetic treatments (n=260, 68.1%). Among those who sought medical aesthetic treatments (n=122, 31.9%), liposuction or fat-dissolving injections were found to be the most preferred treatment (n=18, 26.1%), while skin light and laser treatment was found to be the most favoured non-surgical aesthetic treatment (n=86, 60.6%). A number of platforms were used by the respondents to search for information about medical aesthetic practices including social media (n=145, 20.2%), service providers such as beauty centres (n=110, 15.3%), internet sources such as blogs (n=104, 14.5%), product advertisements (n=93, 12.9%), family or friend referrals (n=90, 12.5%) and newspapers (n=19, 2.6%). Conversely, a minority of the respondents had not actively sought any medical aesthetic treatment-related information (n=158, 22.0%). The respondents’ sociodemographic and treatment characteristics are summarised in Table 1

**Table 1:** Summary of the respondents’ sociodemographic and treatment characteristics.

	Variable	Frequency (%)	Mean ± standard deviation
**Age**			30.81±9.38
**Sex**	Male Female	86 (22.5) 296 (77.5)	
**Marital status**	Single Married Widowed or divorced	242 (63.4) 128 (33.5) 12 (3.1)	
**Ethnicity**	Malay Chinese Indian Others	205 (53.7) 54 (14.1) 106 (27.7) 17 (4.5)	
**Religion**	Muslim Christian Buddhist Hindu Others	218 (57.1) 28 (7.3) 28 (7.3) 108 (28.3) 0 (0)	
**State**	Perlis Kedah Perak Selangor Negeri Sembilan Melaka Johor Pahang Terengganu Kelantan Sarawak Sabah Wilayah Persekutuan Kuala Lumpur Wilayah Persekutuan Putrajaya Wilayah Persekutuan Labuan Penang	2 (0.5) 7 (1.8) 22 (5.8) 203 (53.1) 12 (3.1) 3 (0.8) 19 (5.0) 7 (1.8) 4 (1.0) 5 (1.3) 6 (1.6) 3 (0.8) 64 (16.8) 11 (2.9) 0 (0) 14 (3.7)	
**Location**	City Town Village	289 (75.7) 85 (22.3) 8 (2.0)	
**Highest educational level**	Secondary school Degree Higher degree	23 (6.0) 318 (83.3) 41 (10.7)	
**Occupation**	Student Unemployed/pensioner Employed	119 (31.2) 26 (6.8) 237 (62.0)	
**Income**	<RM 2500 RM 2500-5000 RM 5001-9999 >RM 10,000	156 (40.8) 87 (22.8) 70 (18.3) 69 (18.1)	
Experience in undergoing aesthetic treatment	Yes No	122 (31.9) 260 (68.1)	
**Type of aesthetic treatment performed**	Liposuction or fat-dissolving injection Nose reconstruction (rhinoplasty) Eyelid surgery Breast augmentation (implantation/reduction) Hair transplantation Facelift or thread lift Tummy tuck Buttock lift	18 (26.1) 9 (13.0) 7 (10.0) 8 (11.6) 5 (7.4) 11 (15.9) 5 (7.2) 6 (8.8)	
**Type of non-surgical aesthetic treatment performed**	Any skin lightening and laser treatment Any HIFU skin-tightening procedure Botox, Dysport, Xeomin, etc. Platelet-rich plasma therapy/mesotherapy/microneedling/dermarolling Sclerotherapy or skin blood vessel-shrinking treatment via injection Chemical peeling Microdermabrasion	86 (60.6) 8 (5.6) 9 (6.3) 16 (11.3) 2 (1.4) 16 (11.3) 5 (3.5)	
	<1 year	62 (16.2)
Duration of receiving aesthetic treatment	1–5 years	45 (11.8)
	>5 years	15 (3.9)
	Never	260 (68.1)
	I never search for medical aesthetic treatment	158 (22.0)
	Service provider (e.g. beauty centre)	110 (15.3)
Source of information on aesthetic treatment	Product advertisement	93 (12.9)
	Newspaper Family or friend referral	19 (2.6) 90 (12.5)
	Social media (e.g. Twitter or Facebook)	145 (20.2)
	Internet (e.g. YouTube or blog)	104 (14.5)

The majority of the respondents preferred registered hospitals or clinics over beauty centres to receive the following treatment: dull skin and acne scar treatment (n=242, 63.4%), skin whitening (n=227, 59.4%), wrinkle and scar treatment (n=259, 67.8%), nose or eye reconstructive surgery (n=348, 91.1%) and skin pigmentation laser treatment (n=302, 79.1%). However, beauty centres were the preferred choice for facial cleansing among the majority of the respondents (n=187, 49.0%).

The mean score for the perceptions of the importance of being physically attractive was 30.45±7.97. A total of 206 (53.9%) respondents obtained a mean score of ≥30, representing a positive perception of the importance of being physically attractive. The majority strongly agreed and agreed that they would become more confident (n=97, 25.3%; n=136, 35.6%) and sociable (n=61, 16.0%; n=131, 34.3%); have better job opportunities (n=60, 15.7%; n=122, 32.0%); be more positively perceived by their colleagues (n=59, 15.4%; n=117, 30.6%); be able to find a life partner (n=106, 27.7%; n=55, 14.4%); and be more satisfied (n=88, 23.0%; n=128, 33.5%) if they were physically attractive. Nevertheless, the respondents expressed uncertainty about the longevity of their social relationships (n=174, 45.5%), ease of establishing social relationships (n=165, 43.2%) and social acceptance (n=164, 42.9%) based on their physical attractiveness. The respondents’ perceptions of the significance of being physically attractive are summarised in Table 2.

**Table 2:** Summary of the respondents’ perceptions of the significance of being physically attractive.

Perception: *Being physically attractive or beautiful positively affects me as follows:*	Strongly disagree	Disagree	Neutral	Agree	Strongly agree
I am more confident.	30 (7-9)	11 (2.9)	108 (28.3)	136 (35.6)	97 (25-3)
I am more sociable.	22 (5.7)	16 (4.2)	152 (39.8)	131 (34.3)	61 (16.0)
I believe I have better job opportunities.	23 (6.0)	28 (7.3)	149 (39.0)	122 (32.0)	60(15.7)
I am positively perceived by my colleagues.	24 (6.3)	29 (7-6)	153 (40.1)	117(30.6)	59 (15-4)
I may be able to find a life partner.	37 (9-7)	33 (8.6)	151 (39.5)	106(27.7)	55 (14.4)
I believe my social relationships will last.	36 (9.4)	66 (17.3)	174 (45.5)	71 (18.6)	35 (9.2)
I could build social relationships easily.	28 (7.3)	41 (10.7)	165 (43.2)	106(27.7)	42 (11.1)
I am more socially accepted.	33 (8.6)	26 (6.8)	164 (42.9)	111 (29.1)	48 (12.6)
I am more satisfied with myself.	30 (7-9)	23 (6.0)	113(29.6)	128 (33.5)	88 (23.0)

The mean score for the knowledge of medical aesthetic services was 6.76±2.77. The majority of the respondents obtained mean scores of ≥6 (n=291, 76.2%), representing a good level of knowledge of medical aesthetic services. Most respondents were aware of the requirements to perform medical aesthetic treatments including the necessity for treatment to be administered by a certified aesthetic doctor with an LCP (n=270, 70.7%), the prohibition of such procedures being performed by a beautician or non-medical personnel (n=259, 67.8%) and the need for treatment to be conducted exclusively in a registered clinic or hospital (n=281, 73.6%). Additionally, the majority of the respondents agreed that all advertisements related to medical aesthetics must be approved by the MOH (n=243, 63.6%) and that patients have the right to file a complaint to regulatory bodies upon suspicion of misconduct (n=259, 67.8%). Most respondents expressed their disapproval of the safe application of specific medical aesthetic treatment products and consumables at home such as high-acidity dark spot chemical removal products, hair removal laser devices, chemical peels and intravenous vitamin C drips. The respondents’ knowledge of medical aesthetic practices is summarised in Table 3.

**Table 3:** 

Statement	n (%)		
	True	Not sure	False
Medical aesthetic treatments can be provided by a beautician/non-medical personnel.	44(11.5)	79 (20.7)	259 (67-8)
Medical aesthetic treatments can be provided only by a registered medical practitioner with an LCP from the MOH Malaysia.	270 (70.7)	60(15.7)	52(13-6)
Medical aesthetic treatments can be provided only in registered medical aesthetic clinics or hospitals.	281 (73.6)	53 (13-9)	48(12.6)
It is safe to purchase medical aesthetic items (i.e. botox or dermal fillers) online.	18 (4.7)	62 (16.2)	302 (79.1)
All solutions/chemicals for dark spot removal with high acidity (>20%) are safe to be self-used at home.	15(3-9)	128 (33-5)	239 (62.6)
All hair removal laser devices can be self-used at home.	21 (5-5)	94 (24.6)	267 (69.9)
All chemical peels are safe to be self-used at home.	23 (6.0)	88 (23.0)	271 (70.9)
All intravenous vitamin C drips are considered safe and effective.	49 (12.8)	139 (36.4)	194 (50.8)
All medical aesthetic advertisements need to have approval from medical advertisement boards, including the MOH Malaysia.	243 (63-6)	77 (20.2)	62 (16.2)
As a consumer/client/patient, I can file a complaint directly to regulatory bodies such as the Malaysian Medical Council, MOH Malaysia or Aesthetic Medical Practice Division if I suspect unqualified operators (e.g. beautician/therapist/non-LCP doctor) or unlicensed premises (e.g. beauty centre/medispa/hotel) performing medical aesthetic treatments.	259 (67-8)	78 (20.4)	45(11.8)

LCP, Letter of Credentialing and Privileging; MOH, Ministry of Health

The mean score for the attitudes towards medical aesthetic practices was 27.76±5.08. The majority of the respondents had a mean score of >27 (n=268, 70.2%), representing positive attitudes towards medical aesthetic practices. Most respondents strongly agreed (n=79, 20.7%) and agreed (n=l48, 38.7%) that medical aesthetics contributes to enhancing physical beauty. Conversely, the majority held a neutral stance on the other statements related to aesthetic practices, including views on the potential negative impact of medical aesthetics on health and the belief that aesthetic practices in countries such as South Korea, Thailand or China surpass those in Malaysia. Most respondents expressed uncertainty about whether the side effects of aesthetic treatments are typically severe, irreversible and painful. The respondents were also unsure whether the outcomes of medical aesthetic treatments would meet their expectations or contradict their religious beliefs. The respondents’ attitudes towards medical aesthetic practices are summarised in Table 4.

**Table 4:** Summary of the respondents’ attitudes towards medical aesthetic practices.

Statement	n (%)				
	Strongly disagree	Disagree	Neutral	Agree	Strongly agree
Medical aesthetics helps improve physical beauty.	20 (5.2)	10 (2.6)	125 (32.7)	148 (38.7)	79 (20.7)
Medical aesthetics is harmful to health.	49 (12.8)	95 (24.9)	195 (51.0)	30 (7-9)	13 (3.4)
Medical aesthetic practices are better in other countries such as South Korea, Thailand or China than in Malaysia.	25 (6.5)	39 (10.2)	186 (48.7)	86 (22.5)	46(12.0)
The side effects of aesthetic treatments are usually severe.	27 (7.1)	58(15.2)	206 (53-9)	64 (16.8)	27 (7.1)
The outcomes of medical aesthetic treatments are usually not up to expectations.	14 (3-7)	67(17.5)	229 (59-9)	57 (14.9)	15(3-9)
Medical aesthetic treatments are usually painful.	20 (5.2)	78 (20.4)	204 (53.4)	60 (15.7)	20 (5.2)
Medical aesthetic procedures are expensive in Malaysia.	13(3-4)	38 (9-9)	209 (54.7)	81 (21.2)	41 (10.7)
The effects of aesthetic procedures are usually irreversible.	25 (6.5)	74 (19-4)	213(55.8)	50(13-1)	20 (5.2)
Medical aesthetic procedures contradict my religious beliefs.	39 (10.2)	67 (17.5)	198 (51.8)	43(11-3)	35 (9.2)

Only religion and the perceived significance of being physically attractive were found to have a significant influence on the respondents’ attitudes towards medical aesthetic services. The Buddhists and Hindus were 3.42 and 3.44 times more likely to exhibit a positive attitude towards medical aesthetic services than the Muslims, respectively (Buddhist: odds ratio [OR]=3.4l5, 95% confidence interval [CI]=1.111–10.497, P=0.032; Hindu: OR=3.442, 95% CI= 1.763–6.718, P=0.000). The respondents with higher perception scores on the significance of being physically attractive were 0.5 times less likely to develop a positive attitude towards medical aesthetic services (0R=0.499, 95% CI=0.292–0.850, P=0.011). These results are summarised in Table 5.

**Table 5:** Factors influencing the attitude towards medical aesthetic services.

	Univariate analysis				Binary logistic regression analysis			
	Characteristic	Crude OR	95% CI	Wald’s X^2^ (df)	P	Adjusted OR	95% CI	Wald’s X^2^ (df)	P
**Age**	1.005	0.963-1.048	0.052	0.819	-	-	-	-
**Sex** Male Female	1.00 1.280	0.706-2.322	0.660	0.416				
**Marital status** Single Married Widowed or divorced	1.00 1.232 2.713	0.597-2.543 0.438-16.811	0.318 (1) 1.150 (1)	0.573 0.284	-	-	-	-
**Highest educational level** Secondary school Degree Higher degree	1.00 0.983 0.814	0.352-2.741 0.223-2.965	0.001 (1) 0.097 (1)	0.973 0.755	-	-	-	-
**Occupation** Student Unemployed Employed	1.00 0.783 1.070	0.244-2.507 0.467-2.451	0.170 (1) 0.026 (1)	0.680 0.873	-	-	-	-
**Ethnicity** Malay Chinese Indian Others	1.00 0.517 0.669 0.599	0.086-3.112 0.095-4.710 0.147-2.432	0.519 (1) 0.163 (1) 0.541 (1)	0.471 0.686 0.473	-	-	-	-
**Religion** Muslim Christian Buddhist Hindu	1.00 2.752 7.455 5.004	0.454-16.685 0.932-59.650 0.727-34.439	1.212 (1) 3.584 (1) 2.677 (1)	0.271 0.058 0.102	1.551 3.415 3.442	0.564-4.265 1.111-10.497 1.763-6.718	0.722 (1) 4.597 (1) 13.115 (1)	0.396 0.032 0.000
**Location** City Town Village	1.00 0.625 1.170	0.350-1.116 0.245-5.598	2.529 (1) 0.039 (1)	0.112 0.844	-	-	-	NS
**Income** <RM 2500 RM 2500-5000 RM 5001-9999 >RM 10,000	1.00 0.932 0.510 0.549	0.466-1.864 0.228-1.142 0.214-1.410	0.039 (1) 2.680 (1) 1.553 (1)	0.843 0.102 0.213	-	-	-	NS
**Experience in undergoing aesthetic treatment** Yes No	1.00 0.928	0.268-3.215	0.014 (1)	0.906				
**Duration of receiving aesthetic treatment** <1 year 1-5 years >5 years Never	1.00 2.412 6.079 0.684	0.804-7.234 0.698-52.922 0.202-2.318	2.467 (1) 2.672 (1) 0.373 (1)	0.116 0.102 0.541	2.609 6.895 0.637	0.917-7.428 0.827-57.484 0.341-1.193	3.228 (1) 3.184 (1) 1.985 (1)	0.072 0.074 0.159
**Knowledge score**	1.273	0.742-2.181	0.769 (1)	0.381	-	-	-	-
**Perception score on the importance of being physically attractive**	0.525	0.297-0.927	4.939 (1)	0.026	0.499	0.292-0.850	6.532 (1)	0.011

OR, odds ratio; CI, confidence interval; df, degree of freedom; NS, Non-significance. Income and location were included in the final model based on the significant level (p<0.25) from the univariate test but were found not significant. They were removed from the last step in the final model due to non-significance thus providing no detailed values.

## Discussion

The current study revealed that the majority of the respondents held a positive perception regarding the importance of being physically attractive. They believed that possessing physical attractiveness contributes to enhanced self-confidence and self-satisfaction, consequently fostering positive interpersonal interactions. Regardless of age, physical beauty was perceived as a desirable quality and believed to yield substantial benefits.^13^ Numerous prior studies have highlighted the significance of physical attractiveness, linking it to various outcomes such as higher socioeconomic status, improved behavioural attitudes, enhanced social interactions, 14 increased endurance^15^ and heightened sexual satisfaction.^16^ Moreover, physical attractiveness has been associated with mating and career success, higher educational attainment and increased wages.^6,17,18^ Likewise, western society has also perceived physical attractiveness as an important key social factor associated with desirable traits for leading a fulfilling life.^13,19^ Despite these positive associations, it is crucial to acknowledge that the perception of the importance of physical attractiveness may lead to potential biases, particularly in initial impressions, potentially resulting in unconscious biases.^20,21^

In this study, the respondents were found to have a good level of knowledge related to medical aesthetic services, particularly on legislation processes, which is in line with previous reports in Jordan and Nigeria.^12,22,23^ This could be attributed to the role of several platforms such as social media and internet in providing the public with exposure to relevant information. Therefore, leveraging visually engaging and interactive digital platforms such as Facebook, Instagram and YouTube becomes pivotal for practitioners in disseminating valuable information about medical aesthetics to a wide and accessible audience. Numerous studies have emphasised the value of mass media, including social media (e.g. WhatsApp, Instagram, Twitter and TikTok), television and internet platforms, in disseminating knowledge and news and increasing exposure about medical aesthetic services as well as raising public awareness.^23-25^ However, while it appears that a growing number of people are becoming more informed, a previous study highlighted the prevalence of misunderstandings among patients, largely stemming from false information regarding medical aesthetic procedures.^26^ This misinformation can lead to the development of unfavourable views and distrust towards medical aesthetic services, as individuals may become uncertain and unwilling to seek additional information on the subject.^27^

The majority of the respondents in this study exhibited a favourable attitude towards aesthetic services, agreeing that medical aesthetics helps improve physical beauty. This finding suggests a positive connotation between medical aesthetic services and beauty. According to previous reports, the primary motivation in seeking aesthetic treatments includes increased self-esteem, beautification, positive ageing, transformation and correction, which depend on patients’ personality and sociodemographic characteristics.^20,23^ However, the survey conducted by Salawu et al. among Nigerian internet users exposed negative perceptions towards aesthetic treatments, with respondents expressing hesitancy towards undergoing such procedures.^12^ The study suggested that age, religion, ethnic affiliation and income may contribute to these negative perceptions. Understanding the specific factors influencing negative perceptions within a particular context requires a more in-depth analysis, potentially through qualitative research methods, to capture the nuances and intricacies of individual attitudes and beliefs.

Two factors – perceived importance of physical attractiveness and faith – had a significant influence on the respondents’ attitudes towards medical aesthetic services. The respondents who had higher scores on the perceptions of the importance of being physically attractive were less likely to develop positive attitudes towards medical aesthetic treatments. One possible explanation might be that individuals who place a significant emphasis on the importance of physical attractiveness may have certain reservations or concerns about medical aesthetic treatments. These concerns could include apprehensions of potential risks, fear of unnatural outcomes or cultural influences that shape their views on cosmetic interventions. Furthermore, such individuals might be hesitant to undergo medical aesthetic treatments due to the concern that altering their appearance could result in losing their natural beauty and selfimage. Understanding these underlying factors requires further and more indepth exploration of the concerns held by individuals in the study population.^12^

Herein, the Buddhists and Hindus were more likely to have a favourable perspective towards aesthetic services than the Muslims. This may be because some Muslims may perceive performing aesthetic treatments as a sinful act since these treatments involve altering their physical appearance deliberately. However, the stance on this issue is somewhat ambiguous in Islam.^28^ According to maqasid al-shari’a, which is an Islamic doctrine that promotes the well-being of all humankind while safeguarding their Islamic faith, life, mind, posterity and wealth, aesthetic treatment is permissible as long as the act does not violate general Islamic principles and is not explicitly forbidden in the Al-Quran and hadith typically subjected to Islamic scholars’ fatwa in Malaysia.^29^ In a previous study, it was reported that individuals with a higher degree of devotion or more fervent religious beliefs exhibited a negative attitude and refused to receive medical aesthetic services.^30^

The current study represents the first attempt to explore public perceptions of the perceived importance of being physically attractive and knowledge and attitudes towards medical aesthetic practices in Malaysia. Its contribution to advancing understanding in this specific context may play a role in promoting informed decision-making, enhancing healthcare planning, mitigating risks, improving provider—patient dynamics, ensuring cultural sensitivity and laying the foundation for future developments in the field. Nevertheless, it is important to highlight a few limitations. First, the majority of the respondents were primarily residing in cities, which might have skewed the obtained responses due to differences in the culture, tradition, knowledge and exposure compared with people living in urban areas. Second, the observed disparity in racial representation, particularly the identification of Indians as the second-largest population despite the Chinese being the second-largest ethnic group in Malaysia, might have been influenced by various factors. One potential contributor is the potential variation in the response rates among different ethnic groups, possibly influenced by the fact that the survey distributors were Malay and Indian. Third, since the current study explored only a limited set of factors influencing attitudes towards medical aesthetic services, it emphasises the necessity for a more comprehensive exploration. Acknowledging that the perception of body image is a multifaceted construct influenced by ethical considerations related to societal norms and media influence as well as psychological aspects such as self-perception and mental health underscores the significance of comprehensively understanding and addressing these facets. This comprehensive approach is crucial for promoting a healthier and more positive body image among individuals.

## Conclusion

Most people in Malaysia demonstrate good knowledge and positive attitudes towards medical aesthetic treatments despite having no prior experience. This suggests a growing interest and potential willingness to consider medical aesthetic treatments as options for enhancing appearance. Educational campaigns and promotions should be further enhanced to disseminate reliable and trustworthy information to the public. This would help dispel any uncertainties and misconceptions related to medical aesthetic practices among the general public. Future studies should adopt a more comprehensive approach, exploring the multifaceted nature of body image perceptions by delving into the intricate interplay of ethical considerations tied to societal norms and media influence.

## Data sharing statement

Data are available only upon request, as they contain private information that could violate the data privacy of the respondents.

**How does this paper make a difference in general practice?**

- The findings of this study help enhance the understanding of public awareness regarding medical aesthetic services, contributing to the development of these services both locally and globally.
- The study not only identifies the factors influencing public awareness of medical aesthetics but also provides valuable insights for regulatory bodies and medical institutions to formulate strategies aimed at improving public awareness and perception of medical aesthetic services.
